# TAI CHI OR CONVENTIONAL EXERCISE ON PHYSICAL PERFORMANCE, SLEEP, AND QUALITY OF LIFE IN OLDER PEOPLE WITH DEMENTIA

**DOI:** 10.1093/geroni/igac059.1813

**Published:** 2022-12-20

**Authors:** Ran Gao, Chieko Greiner, Hirochika Ryuno, Shuhong Ｇao

**Affiliations:** Kobe University, Kobe, Hyogo, Japan; Kobe University, Kobe-city, Hyogo, Japan; Kobe University, Kobe city, Osaka, Japan; Hainan nursing home, Haikou city, Hainan, China (People's Republic)

## Abstract

**Aims:**

This study was to compare the therapeutic efficacy of Tai chi and conventional exercise on physical performance, sleep efficiency and quality of life among older people with dementia.

**Methods:**

Eighty-one elderly mild to moderate older people with dementia living in a nursing home in China were screened and randomized to three groups. The intervention groups received a 12-week Tai chi or conventional exercise intervention. Participants were assessed at baseline, week 6, and week 12 between December 2020 and July 2021. The primary outcome was short physical performance battery, secondary outcomes including the sleep efficiency and quality of life. Generalized estimating equations and one-way analysis of variance were the main statistical tests.

**Results:**

The mean age was 80.7 (SD 7.7) years and 74.1% of participants were women. Analysis indicated that there was a significant difference in both intervention groups in short physical performance battery, sleep efficiency and quality of life relative to control from 0-12 weeks, and the Tai chi group had a substantial advantage over the conventional exercise group.

**Conclusions:**

Tai chi and conventional exercise could improve physical performance, sleep efficiency and quality of life in older people with dementia. Furthermore, Tai chi could be a more effective approach to improve physical performance.

